# Traces of JC polyomavirus in papillary thyroid cancer: a comprehensive study in Iran

**DOI:** 10.1186/s12985-022-01881-4

**Published:** 2022-09-26

**Authors:** Amir Ali Karimi, Rahil Tarharoudi, Zahra Kianmehr, Fatemeh Sakhaee, Fatemeh Rahimi Jamnani, Seyed Davar Siadat, Abolfazl Fateh

**Affiliations:** 1 Department of Biotechnology, Faculty of Biological Science, North Tehran Branch, Islamic Azad University, Tehran, Iran; 2 Department of Molecular and Cellular Biology, Faculty of Advanced Science and Technology, Tehran Medical Sciences, Islamic Azad University, Tehran, Iran; 3 Department of Biochemistry, Faculty of Biological Science, Islamic Azad University, North Tehran Branch, Tehran, Iran; 4 Department of Mycobacteriology and Pulmonary Research, Pasteur Institute of Iran, Tehran, Iran; 5 Microbiology Research Center (MRC), Pasteur Institute of Iran, Tehran, Iran

**Keywords:** JCPyV, Papillary thyroid cancer, Non-cancerous sample

## Abstract

**Background:**

JC polyomavirus (JCPyV) is known to induce solid tumors such as astrocytomas, glioblastomas, and neuroblastomas in experimental animals, and recent studies have shown that the virus may be correlated with carcinogenesis. This study aimed to evaluate the impact of JCPyV on the progression of papillary thyroid cancer (PTC).

**Methods:**

A total of 1057 samples, including 645 paraffin-embedded PTC biopsy samples (PEBS) and 412 fresh biopsy samples (FBS), and 1057 adjacent non-cancerous samples were evaluated for the presence of JCPyV DNA and RNA.

**Results:**

We observed that 10.8% (114/1057) samples, including 17.5% (72/412) FBS and 6.5% (42/645) PEBS were positive for the JCPyV DNA. Among the JCPyV-positive samples, the mean JCPyV copy number was lower in patients with PEBS (0.3 × 10^–4^ ± 0.1 × 10^–4^ copies/cell) compared to FBS (1.8 × 10^–1^ ± 0.4 × 10^–1^ copies/cell) and non-PTC normal samples (0.2 × 10^–5^ ± 0.01 × 10^–5^ copies/cell), with a statistically significant difference (*P* < 0.001). The *LT*-Ag RNA expression was lower in PEBS than in FBS, while no *VP1* gene transcript expression was found.

**Conclusions:**

Although our results confirmed the presence of JCPyV in some Iranian patients with PTC, more research is needed to verify these results.

## Background

Thyroid cancer (TC) is the ninth most common cancer in the world, and its incidence has increased dramatically over the last 40 years. In 2020, 586,202 new cases of TC occurred, with papillary TC (PTC) accounting for more than 80% of all TCs [1]. Although individuals might gain PTC at any age, most patients come to the hospital before 40. Risk factors for PTC include exposure to radiation and a family history of TC. However, it should be noted that most patients have no risk factor at all [2]. The correlation of TC with human parvovirus B19, BK polyomavirus (BKPyV), human herpes simplex virus, Epstein–Barr viruses, hepatitis C virus, and Merkel cell polyomavirus (MCPyV) has been evaluated and confirmed by several studies [3, 4].

John Cunningham virus (JCPyV) is a polyomavirus and a DNA virus. Seroepidemiological reports have shown that asymptomatic primary infections with JCPyV happen in childhood under the age of 5 years**,** and approximately 85% of the population will be seropositive by adulthood. After the primary infection, JCPyV hides in diverse regions, such as the kidneys, bone marrow, and tonsils. However, it is not clear whether central nervous system (CNS) latency will occur. Reactivation of JCPyV in immunocompetent patients commonly takes the form of asymptomatic viruria [5].

Progressive multifocal encephalopathy (PML) is a deadly demyelinating illness of the CNS caused by the JCPyV. PML is a scarce disease that is most frequently associated with people who have an underlying immunosuppressive condition, such as acquired immunodeficiency syndrome (AIDS), lymphoproliferative disorders, Hodgkin's lymphoma, or who are receiving anticancer medication [6].

Polyomavirus genomes can be classified into three regions, which are as follows: the early region that named T-antigens (T-Ag), which encodes early proteins; the control region; and the late region, which contain late proteins with structural function. The early genes encode proteins that regulate viral transcription and genome replication. The viral transforming potential is directly linked to T-Ag expression. One of the T-Ags is large T (*LT*)-Ag that is a nuclear protein with about 700 amino acids. However, changes in its phosphorylation pattern may result in a shift in the protein's position inside the cell. Both viral transcription and genome replication are regulated by the *LT*-Ag. Additionally, the LT-Ags and other T-Ag isoforms expressed in human and animal polyomaviruses are pleiotropic proteins that influence the activity and expression of various cellular proteins involved in cell proliferation regulation [7–9].

DNA and proteins of JCPyV have been identified in a broad range of glial and non-glial human cancers, such as medulloblastomas, gliomas, and ependymomas, and several non-glial clinical samples of lower and upper gastrointestinal cancers [10, 11]. These data indicate that JCPyV can infect various cell types, but its role in human carcinogenesis remains unclear. Therefore, this study aimed to determine if JCPyV is involved in PTC.

## Methods

### Patients and samples collection

The present study was carried out at the Pasteur Institute of Iran during October 2014-March 2020 according to the Declaration of Helsinki (1975) and local regulations. The study was also approved by the Ethics Committee of Pasteur Institute of Iran, and written informed consent was directly obtained from the patients.

A total of 1057 biopsy samples, including 645 paraffin-embedded PTC biopsy samples (PEBS) and 412 fresh biopsy samples (FBS), and 1057 adjacent non-PTC normal samples were collected from three centers in Tehran, Iran.

### DNA and RNA extraction

Deparaffinization was performed, as previously described [4]. After tissue deparaffinization, genomic DNA from FBS and PEBS was extracted using a High Pure FFPET DNA Isolation Kit (Roche Diagnostics Deutschland GmbH, Mannheim, Germany), according to the manufacturer’s instructions**.**

Total RNA from the tissue sample sections was extracted with the RNeasy Kits (QIAGEN, CA, USA), according to the manufacturer’s instructions.

### JCPyV detection by conventional PCR

In order to confirm the integrity of DNA, the β-globin gene was amplified. Polymerase chain reaction (PCR) was carried out with 500 ng of DNA by the PCR master mix 2x (SinaClon BioScience, Tehran, Iran) and 0.5 μM of each primer in a total volume of 25 μl. For identifying the presence of the JCPyV, large T antigen (*LT-Ag*) gene with the size of 248 bp was used, as described previously [12]. The QIAquick PCR Purification Kit (Qiagen, Hilden, Germany) was used for the PCR products purification, according to the manufacturer’s instructions. Then, ABI automated sequencer (Applied Biosystems, Foster City, CA, USA) was applied for PCR products sequencing. The raw sequencing data were analyzed by MEGA version 6.0 software (http://www.megasoftware.net).

### Measurement of JCPyV DNA load

The JCPyV DNA load was measured by LightCycler® 96 Real-Time PCR System (Roche Diagnostics Deutschland GmbH, Mannheim, Germany) with the PCR program, primers, and probe sequences that were previously described [13, 14]. The JCPyV DNA load was determined by dividing the JCPyV DNA copy number by half of the RNase P gene copy number (each diploid cell had two copies of the RNase P gene). Plasmids for JCPyV *LT*-Ag and human RNase P gene for drawing a standard curve for real-time PCR were created based on previous studies [13, 14]. Adjacent normal non-PTC specimens were tested to eliminate the possibility of contamination and false positive results.

### Measurement of JCPyV RNA expression

The transcripts of *LT*-Ag and viral protein (VP1) genes were assessed using qualitative real-time reverse transcription PCR (real-time RT-PCR) with primers and PCR program previously described [15]. RNA extracted from progressive multifocal leukoencephalopathy (PML) lesion was used as a positive control. Melting curve analysis was performed on the PCR products to confirm the specificity of amplification.

### Statistical analysis

A statistical analysis was completed via the SPSS software version 22.0 (SPSS Inc., Chicago, IL, USA). To test for normality of distribution of continuous data, we applied the Shapiro–Wilk test. Quantitative and continuous variables were also evaluated using Pearson's Chi-square and Mann–Whitney U tests, respectively. Two-tailed *P* < 0.05 was defined as statistically significant [16].

## Results

### Demographic and clinical characteristics of patients

The clinical characteristics and baseline demographics of the patients included in the study are summarized in Table 1. In brief, out of 1057 assessed patients with PTC, 275 (26.0%) subjects were male, and 782 (74.0%) cases were female. The mean age and tumor size were 42.5 ± 13.1 years and 1.9 ± 1 mm, respectively. In total, 47.5% (502/1057) patients, including 262 male and 240 female participants, were a smoker.

**Table 1:** Demographic features in Iranian Patients with papillary thyroid cancer according to JCV *LT*-Ag positivity

	JCV *LT*-Ag positivity		Total	*P*-value
	Positive	Negative		
Patients (No)	114 (10.8%)	943 (89.2%)	1057 (100.0%)	–
Mean age ± SD	40.7 ± 13.3	42.8 ± 13.1	42.5 ± 13.1	0.065
Sex (male/female)	44/70 (38.6/61.4%)	231/712 (24.5/75.5%)	275/782 (26.0/74.0%)	0.001*
Smoking (yes/no)	72/42 (63.2/36.8%)	430/513 (45.6/54.4%)	502/551 (47.5/52.5%)	0.275
Tumor size (mean ± SD, mm)	2.1 ± 1.3	1.9 ± 0.9	1.9 ± 1.0	0.448
Multifocality (unifocal/multifocal)	47/67 (41.2/58.8%)	621/322 (65.9/34.1%)	668/389 (63.2/36.8%)	< 0.001*
Lymphovascular invasion (yes/no)	66/48 (57.9/42.1%)	128/815 (13.6/86.4%)	194/863 (18.4/81.6%)	< 0.001*
Extrathyroidal extension (yes/no)	67/47 (58.8/41.2%)	93/850 (9.9/90.1%)	160/897 (15.1/84.9%)	< 0.001*
Lymph node involvement (yes/no)	71/43 (62.3/37.7%)	334/609 (35.4/64.6%)	405/652 (38.3/61.7%)	< 0.001*
Capsular invasion (yes/no)	70/44 (61.4/38.6%)	431/512 (45.7/54.3%)	501/556 (47.4/52.6%)	0.002*

*SD* standard deviation, *mm* millimeter

*Statistically significant (< 0.05)

### JCPyV genome detection by conventional PCR

Among 1057 tested patients, 412 (39.0%) and 645 (61.0%) samples were FBS and PEBS, respectively. The presence of JCPyV in 1057 PTC and 1057 adjacent non-PTC normal cells was tested by *LT*-Ag primer using conventional PCR. The JCPyV DNA was identified in 10.8% (114/1057) samples. In addition, it was detected in 72 (17.5%) of 412 FBS and 42 (6.5%) of 645 PEBS. In order to confirm the results of conventional PCR, the purified PCR products were sequenced. This sequence showed 100% homology with another Iranian isolate, JCPyV-*LT*-Ag (GenBank: KJ719314.1).

Among 1057 adjacent non-PTC normal samples, the JCPyV DNA was detected in 0.7% (3/1057) FBS. The β-globin gene was consistently amplified in all samples.

### Comparison of JCPyV DNA load in FBS and PEBS

In the current study, the PTC samples positive by conventional PCR were chosen for quantitative real-time PCR to assess the DNA load. Out of 1057 samples, the JCPyV *LT*-Ag was found in 114 (10.8%) of patients, 38.6% (44/114) and 61.4% (70/114) of whom were male and female, respectively. The JCPyV positivity was significantly different between sex (*P* = 0.001), extrathyroidal extension (*P* < 0.001), lymphovascular invasion (*P* < 0.001), lymph node involvement (*P* < 0.001), capsular invasion (*P* = 0.002), and multifocality (*P* < 0.001).

Overall, JCPyV *LT*-Ag DNA load was quantified in 17.5% (72/412) and 6.5% (42/645) of FBS and PEBS, respectively. The JCPyV *LT*-Ag positivity in FBS compared to PEBS was significantly different between extrathyroidal extension (*P* < 0.001), lymphovascular invasion (*P* < 0.001), lymph node involvement (*P* = 0.038), and capsular invasion (*P* = 0.002; Table 2).

**Table 2:** Comparison of demographic features between fresh and paraffin-embedded papillary thyroid cancer biopsy samples

	Fresh biopsy samples (n = 412)	Paraffin-embedded biopsy sample (n = 645)	*P*-value
Mean age ± SD	42.7 ± 13.4	42.5 ± 12.9	0.065
Sex (male/female)	117/295 (28.4/71.6%)	158/487 (24.5/75.5%)	0.172
Smoking (yes/no)	113/299 (27.4/72.6%)	389/256 (60.3/39.7%)	0.472
Tumor size (mean ± SD, mm)	2.1 ± 1.2	1.9 ± 0.9	0.065
Multifocality (Unifocal/Multifocal)	248/164 (60.2/39.8%)	420/225 (65.1/34.9%)	0.117
Lymphovascular invasion (yes/no)	102/310 (24.8/75.2%)	92/553 (14.3/85.7%)	< 0.001*
Extrathyroidal extension (yes/no)	84/328 (20.4/79.6%)	76/569 (11.8/88.2%)	< 0.001*
Lymph node involvement (yes/no)	174/238 (42.2/57.8%)	231/414 (35.8/64.2%)	0.038*
Capsular invasion (yes/no)	232/180 (56.3/43.7%)	269/376 (41.7/58.3%)	< 0.001*
JCV (positive/negative)	72/340 (17.5/82.5%)	42/603 (6.5/93.5%)	< 0.001*
JCV DNA load (mean ± SD)	1.8 × 10^–1^ ± 0.4 × 10^–1^	0.3 × 10^–4^ ± 0.1 × 10^–4^	< 0.001*

*SD* standard deviation, *mm* millimeter

*Statistically significant (< 0.05)

In the JCPyV-positive samples, the mean JCPyV copy number was significantly higher in FBS (1.8 × 10^–1^ ± 0.4 × 10^–1^ copies/cell) than PEBS (0.3 × 10^–4^ ± 0.1 × 10^–4^ copies/cell) (*P* < 0.001; Table 2). In adjacent non-PTC normal specimens, the mean JCPyV copy number was lower than the cancerous samples (0.2 × 10^–5^ ± 0.01 × 10^–5^ copies/cell).

### Comparison of JCPyV *LT*-Ag RNA Expression between FBS and PEBS

Measuring the viral DNA load alone was insufficient to study the relationship between JCPyV positivity and PTC. Therefore, *LT*-Ag and *VP1* genes expression in JCPyV DNA-positive patients was assessed at the JCPyV *VP1* and *LT*-Ag RNA level by real-time RT-PCR. Out of 114 samples positive for JCPyV DNA, all FBS and 45.2% (19/42) PEBS were suitable for RNA extraction. We found that 61 (84.8%) of 72 FBS and only 6 (31.6%) of 19 PEBS expressed *LT*-Ag RNA. The expression of *LT*-Ag RNA in FBS (2.3 × 10^–2^ ± 0.5 × 10^–2^ copies/cell) was higher than in PEBS (0.6 × 10^–5^ ± 0.2 × 10^–5^ copies/cell) (*P* < 0.001). No *VP1* gene transcript expression was found.

## Discussion

The viral etiology of human cancers is an interesting topic, and to date, several virus types have been identified in human cancers. JCPyV infects most of the world's population, with 80%-90% of adults being serum-positive [17]. Cell infection by JCPyV has two possible outcomes, including lytic infection in permissive cells that allows viral DNA replication and oncogenesis in nonpermissive cells, which do not support viral DNA replication resulting in incomplete infection or malignant cell transformation [18]. Previous studies indicated a feasible role of JCPyV in several cancers, namely brain cancers of medulloblastomas and glial origin, as well as epithelial cancers, such as colon, prostate, breast, and lung adenocarcinoma, and esophageal carcinomas [10, 13, 17, 19, 20].

To the best of our knowledge, the current study was the first that assessed the correlation between JCPyV infection and PTC based on pathological characteristics. Few studies have investigated the relationship between polyomaviruses and TC. Three of those studies found the sequence of simian vacuolating virus 40 (SV40), and the other investigations reported MCPyV and BKPyV in post-operative thyroid samples [4, 21–24].

The four Koch postulates have not been fulfilled by any of the oncogenic viruses discovered to date. It remains controversial whether polyomaviruses can cause TC. Austin Bradford Hill proposed broader epidemiological criteria for causality, which have become accepted and can be used when assessing potential virus-cancer associations. These criteria are based on relevance strength, consistency, analogy, specificity, transient, biological validity, consistency with known factors, and experimental validation [25, 26]. It has been proposed that genome integration instead of just identifying viral sequences or proteins may provide a deeper understanding of the link between viruses and cancer. More convincing evidence is certainly needed to confirm the latter hypothesis [27]. The current research is only the beginning of a lengthy path to determine whether these viruses cause TC or are simply innocent bystanders. However, we indicated the correlation between JCPyV positivity and PTC.

We compared the JCPyV positivity and DNA load between PEBS and FBS in the current study. The results of our study revealed that JCPyV DNA existed in 17.5% and 7.5% of FBS and PEBS, respectively. Moreover, the mean JCPyV DNA load was significantly lower in patients with PEBS than FBS. It seems that FBS has been appropriate for detecting viral infection in PTC patients. In comparison to PEBS, the process of FBS is much faster. While PEBS is insufficient for molecular analysis, FBS is ideal for this purpose. This is due to the PEBS preparation, which impacts the molecular data. In techniques such as next-generation sequencing, mass spectrometry, and quantitative real-time PCR, FBS is recommended. As a result, these samples are considered the gold standard for DNA and RNA analysis [4, 28].

The lack of identification of JCPyV *LT*-Ag DNA and RNA in most positive PEBS, as well as the low amount of DNA copies per cell, could be attributed to a variety of factors. First, DNA and RNA degradation occur in PEBS, reducing DNA and RNA yields in conventional and real-time PCR analysis [29]. Second, the low copy of viral DNA and RNA in PEBS could indicate that JCPyV is present as a passenger virus in these samples without any clinical symptoms. Third, JCPyV may have a role in cancer etiology through a "hit-and-run" mechanism [30]. Viral hit-and-run oncogenesis scenarios suggest that temporary oncoviruses genome acquisition can cause a permanent change in the gene expression pattern of the host cell, leading to malignant transformation [29]. In this case, viral genomes or tiny viral fragments may be discovered in malignant tumors or tumor precursors. Ironically, both transformational activities and hit-and-run processes have been investigated in non-transforming viruses that appear to be unrelated to human cancer [4, 31].

JCPyV DNA positivity alone is insufficient for determining the etiology of JCPyV as another infectious agent linked to PTC [4]. Several studies have demonstrated that polyomaviruses *LT*-Ag expression is needed for the carcinogenesis [4, 29].

In the present study, the JCPyV *LT*-Ag DNA loads were lower in non-cancerous and cancerous cells samples. Several reports in different types of cancer indicated that the frequency of polyomaviruses DNA was significantly lower in the cancerous tissues than tumor cells [4, 29]. This finding suggests a transient role for polyomaviruses in cell transformation because their genome can be silenced or destroyed during cancer progression, which points out the hit-and-run mechanism [32].

According to this theory, we assessed the expression of the *LT*-Ag gene at the RNA level. In this study, 84.8% of FBS and 31.6% of PEBS expressed *LT*-Ag RNA, while *VP1* gene transcript was not found in any of the samples. Similar to JCPyV, polyomaviruses have an ordered gene expression cascade in which the *LT* gene transcript is expressed first as early gene transcription, followed by the *VP1* gene expression as of late gene transcription [4]. The lack of viral replication ability, on the other hand, is a common hallmark of virus-related malignancies [33]. For example, MCPyV replication in most merkel cell carcinoma is disrupted, with only the truncated *LT* gene but not the *VP1* gene being permanently expressed and the viral DNA integrated [34, 35]. Considering the mentioned results, it is logical that we only detected *LT* RNA expression in PTC samples, but not the *VP1* gene RNA expression that reflects viral DNA replication.

In this regard, several reports evaluated *LT*-Ag expression and localization utilizing the immunohistochemistry technique. The localization of effective immunoreactivity inside the tumor cell nucleus indicated exclusive *LT*-Ag expression [36, 37]. One of the limitations of the present research was the lack of immunohistochemistry.

## Conclusion

In the current study, JCPyV DNA and RNA transcripts were found in PTC for the first time. Furthermore, our results demonstrated that the viral genome load in PEBS was lower than in FBS. However, further epidemiological and virological studies in different parts of the world are required to determine the relationship between JCPyV pathogenicity and PTC.

## Data Availability

All data generated or analyzed during this study are included in this article. In addition, the sequence has been deposited to GenBank database with the accession number of OM925538.

## Abbreviations

JCPyV: JC polyomavirus
PTC: Papillary thyroid cancer
PEBS: Paraffin-embedded PTC biopsy samples
FBS: Fresh biopsy samples
TC: Thyroid cancer
MCPyV: Merkel cell polyomavirus
PCR: Polymerase chain reaction
*LT-Ag*: Large T antigen
VP1: Viral protein
PML: Progressive multifocal leukoencephalopathy
Real-time RT-PCR: Real-time reverse transcription PCR
SV40: Simian vacuolating virus 40
